# Could Some Geriatric Characteristics Hinder the Prescription of Anticoagulants in Atrial Fibrillation in the Elderly?

**DOI:** 10.1155/2014/693740

**Published:** 2014-09-10

**Authors:** Paule Denoël, Jacques Vanderstraeten, Pierre Mols, Thierry Pepersack

**Affiliations:** ^1^Emergency Service, Europe Hospital, 1040 Brussels, Belgium; ^2^Centre de Recherche en Santé Environnementale et en Santé du Travail, Ecole de Santé Publique, Université Libre de Bruxelles, 1070 Brussels, Belgium; ^3^Emergency Service, Saint-Pierre University Hospital, Université Libre de Bruxelles, 1000 Brussels, Belgium; ^4^Department of Geriatrics, Erasme Hospital, Université Libre de Bruxelles, Lennik Street 808, 1070 Brussels, Belgium

## Abstract

Several studies have reported underprescription of anticoagulants in atrial fibrillation (AF). We conducted an observational study on 142 out of a total of 995 consecutive ≥75 years old patients presenting AF (14%) when admitted in an emergency unit of a general hospital, in search of geriatric characteristics that might be associated with the underprescription of anticoagulation therapy (mostly antivitamin K at the time of the study). The following data was collected from patients presenting AF: medical history including treatment and comorbidities, CHADS_2_ score, ISAR scale (frailty), Lawton's scale (ADL), GDS scale (mood status), MUST (nutrition), and blood analysis (INR, kidney function, and albumin). Among those patients for who anticoagulation treatment was recommended (73%), only 61% were treated with it. In the group with anticoagulation therapy, the following characteristics were observed more often than in the group without such therapy: a recent (≤6 months) hospitalization and medical treatment including digoxin or based on >3 different drugs. Neither the value of the CHADS_2_ score, nor the geriatric characteristics could be correlated with the presence or the absence of an anticoagulation therapy. More research is thus required to identify and clarify the relative importance of patient-, physician-, and health care system-related hurdles for the prescription of oral anticoagulation therapy in older patients with AF.

## 1. Introduction

Atrial fibrillation (AF) is the most frequent chronic arrhythmia in the elderly, affecting up to 10% of the population aged 80 and above. With the population aging, the burden of AF is expected to double over the next two generations, making this arrhythmia an increasingly important public health issue [1–4]. The risk of thromboembolic complications (stroke and systemic embolism) is high in AF, with the relative risk of ischemic stroke being evaluated to about five in AF without mitral valvular disease [5]. The indication of anticoagulation therapy with antivitamin K (AVK) for the prevention of thromboembolism in patients with AF is based on information derived from numerous well-designed and randomized control trials [6, 7].

Numerous studies have notably shown that long term anticoagulation treatment with AVK reduces the risk of ischemic stroke in patients with nonvalvular AF by about 68% [8, 9]. As a comparison, this reduction is only 22% for acetylsalicylic acid (ASA) at preventive doses (75 to 325 mg/day) [10, 11], with a hemorrhagic risk that is otherwise not lower than the one for AVK [12, 13].

In spite of current evidence of the benefits of anticoagulation therapy for AF in patients with moderate to high CHA_2_DS_2_-VASc risk scores [14, 15], AVK is underprescribed as highlighted in this study of the elderly (from here on defined as corresponding to the age of 75 and above).

While 50 to 60% of geriatric patients with AF should be treated with an AVK, only 15 to 44% are actually treated with it [16–18]. The studies evaluating possible hurdles to the prescription of AVK reveal three distinct types of obstacles: patient-, physician-, and health care system-related factors [18]. Physician-related difficulties reflect their misconception of the risk/benefit ratio of administering oral anticoagulation therapy. Within this category, we have identified three elements that may influence the prescriber. First, the personal experience of the prescriber such as a negative event with a patient (major gastrointestinal or cerebral bleeding) might discourage the prescription of AVK [16]. The second element is linked to the patients' own attributes. Some psychosocial factors may discourage the prescription of anticoagulants because of the concern of a higher risk of hemorrhagic complications [19] due to poor compliance. These factors are related to drug or alcohol abuse, psychiatric pathologies (schizophrenia, psychosis, major depression, etc.), underprivileged situations (patient isolated or without a fixed domicile) and/or a history of noncompliance. Other determining patients' attributes are the particulars of the geriatric patient, aged ≥75, and the specific risk of a potential fall, unjustifiably discouraging the prescription of AVK [16, 20–22], or inciting to target INR (international normalized ratio) levels that are below 2.0 [16, 20]. The third element of the misperception of the risk versus benefit ratio of AVK experienced by the prescriber is the constraints (INR control) and contingencies (food interactions, bleeding risk) of oral anticoagulation therapy [23, 24] and the complexity of keeping INR within the therapeutic range (2.0-3.0) [16, 18, 24, 25]. According to available data, INR remains within this range only 40 to 58% of time [26, 27].

The recipient's perception of the benefit versus risk ratio of AVK therapy also influences the agreement to submit to anticoagulation therapy. Well informed patients are more prone to accept the risk of such treatment [28]. One study reported that among those patients treated with AVK for AF, 48% did not understand the purpose of the treatment and 37% were not aware of their own risks of stroke [2]. The information provided to the patient also influences patient's decision to accept an AVK treatment [28]. Another study reported that the percentage of patients accepting AVK treatments varied between 30 and 87%, depending on the prescriber and the way he delivered such information about the treatment [29]. In general, patients appear to accept a lower benefit/risk ratio than the prescribers do [30, 31]. Finally, some health care system-related factors have also been identified as hindering AVK prescription in AF [18]; one example refers to the availability of a nearby laboratory for INR measurements [32].

It should be further investigated whether some geriatric characteristics or geriatric score could be predictive of the decision of the prescriber for anticoagulation in AF in the elderly. One study [33] compared some geriatric attributes of patients with AF who were given anticoagulants with those with AF who were not. This study was retroactive and was achieved in the geriatric unit of a general hospital. The following parameters were evaluated: CHADS_2_ score, Katz score, Lawton scores (daily life activity), and various geriatric characteristics (cognitive, nutrition and mood status, and risk of fall). No difference could be observed between the two patients groups. Here, we suggest searching again for such predictive geriatric characteristics in patients ≥75 years old, this time in a prospective way, within the context of an emergency room while increasing the number of features to be investigated.

## 2. Objective

The aim of this study was to observe the prevalence of (nonvalvular) AF in consecutive geriatric patients admitted in an emergency room and to analyze the variables associated with such underprescription of anticoagulation therapy in patients with AF.

## 3. Methods

### 3.1. Design

This was an observational study. Consecutive eligible patients were assessed by the team in the emergency room under the supervision of one physician (first author).

### 3.2. Setting

The study took place in an emergency unit of a general hospital.

### 3.3. Patients

All consecutive patients ≥75 years old admitted between 27 May 2011 and 23 December 2012 in the emergency unit were assessed for the presence of (nonvalvular) AF. The total number of admitted patients as well as the number of the patients with AF was collected during the length of the study. The diagnosis of AF was based on the reading of a 12-lead-ECG. Patients with a history of paroxysmal AF were not enrolled in this study. Comprehensive assessment covered only those patients with AF irrespective of the presence or not of AVK (or low molecular weight heparin) therapy. Patients who were not able to answer the questionnaires of the comprehensive assessment were excluded and their number collated. Exclusion criteria were the following: language other than French, English or Dutch, aphasia, critical condition, and/or obvious cognitive disorders.

### 3.4. Comprehensive Assessment

The following items were systematically gathered and recorded.

- The cause of admission (general status alteration, fall, confusion, traumatism, dyspnea, precordium pain, stroke suspicion, pyrexia, and other).
- A social evaluation stating the age, residency status (private home versus institution), living situation (alone or not), and presence (or absence) of a followup by a general practitioner.
- The patient's medical history, with a special emphasis on previous history of stroke or transitory ischemic attack (TIA).
- The medical treatment (drugs administrated to the patient before admission), with special attention to AVK therapy.
- The risk of an embolic stroke and related level of indication for anticoagulation therapy according to the CHADS_2_ score [34].
- The frailty of the patient, using the tool for* Identification of Senior at Risk *(ISAR) [35].
- The level of daily activities (ADL) using the Lawton's scales [36], with special focus on the ability to manage medications.
- The determination whether the patient had previous incidences of falling (≥1 fall within the 6 months before admission: named “faller”) or not.
- The probability of depression using the geriatric* depression* scale (GDS) [37].
- The nutritional status using the* Malnutrition Universal Screening Tool* (MUST) [38].
- A polypathology and severity of the medical problems using the Charlson's comorbidity index [39].
- The determination of the levels of serum urea, creatinine, albumin, and INR.

The dispatch of the patients were recorded (hospitalization or not). The use of the CHA_2_DS_2_-VASc instead of the CHADS_2_ score would not have significantly changed the proportion of patients eligible for an anticoagulation therapy in our patient sample. We also choose not to determine the HAS-BLED score, because this score is hardly, if ever, used by general practitioners in our country; moreover, several criteria of this score (hypertension, stroke, and age) are also included in the CHADS_2_ score and some (renal insufficiency, drugs) do not show any contraindication for AVK treatment.

### 3.5. Ethical Committee

All the research for this study met the criteria of the routine Good Clinical Practices. Data from the medical charts were collected anonymously in a database.

### 3.6. Statistics

Results were collected in a database on Microsoft's Excel and statistical analyses were performed using Stata 11.2 software (Lakeway Drive, Texas, United States). Results are means (±1 SD). Unpaired Student's *t*-test, comparing means and squared chi, was used to assess between group differences in proportion of variables. Univariate and multivariate logistic regression analyses were achieved in order to identify the variables that are associated with the anticoagulated status. A *P* value < 0.05 is considered as statistically significant.

## 4. Results

Between May 27th 2011 and December 23rd 2011, 1204 patients ≥75 years old were admitted at the emergency unit. Among those patients, 92 presented exclusion criteria and 117 were excluded because data collected was incomplete.

Among the 995 retained patients, 142 presented with atrial fibrillation (14%).  Table 1 illustrates the characteristics of this group.

Among patients with AF for whom anticoagulation was recommended (73% of all patients), only 61% actually received anticoagulation therapy.

The main cause of their admission at the emergency room was “a deterioration of general status.” Four percent of the patients were admitted for suspicion of a stroke and/or transient ischemic attack (TIA). None of those patients revealed a cerebral hemorrhage by brain CT-scan, and only 1 of those patients received AVK therapy (INR of 2.4 at the time of his admission).

Among those patients admitted after trauma (with or without a fall) and those admitted after a fall (30 patients, 15 receiving AVK therapy), only one patient presented a cerebral hemorrhage. This last patient had not received any AVK or aspirin therapy before admission.

In order to analyze geriatric characteristics potentially associated with the underprescription of AVK in AF, we compared geriatric characteristics, comorbidities, ISAR score, and drug treatment between patients receiving anticoagulation therapy and patient not receiving that therapy (Table 2): higher proportions of patients with recent (≤6 months) hospitalization, comedication with digoxin, and taking >3 different drugs were observed in the group receiving anticoagulation therapy than in the group not receiving it. All other parameters were not significantly different between these two groups. Comparing the percentages of patients receiving anticoagulation therapy according to the presence of digoxin in the admission treatment, we observed that 85% of the patients receiving this drug also received anticoagulation therapy, while only 55% of the patients without digoxin treatment received it (*P* < 0.003).

In univariate analysis, no geriatric characteristic was associated with the presence (or the absence) of anticoagulation therapy (Table 3). In multivariate analysis, only the presence of previous hospitalization within the last 6 months and the presence of digoxin treatment were factors associated with anticoagulation therapy (Table 4). The goodness-of-fit of this model remains significative, suggesting that those two factors were probably confounding.

## 5. Discussion

To the best of our knowledge, the present study is the first of its kind to address the issue of the variables associated with the underprescription of AVK in AF in the elderly, in a prospective way and in an emergency service. Out of a total of 995 geriatric subjects, the study covered 142 patients with AF. At first glance, one should notice factors which limit a potential generalization of the results. To begin, it concerned only a relatively small number of subjects in the context of their admission in the emergency service of a single, general, hospital that is situated in a neighborhood with a relatively high standard of living and where most patients have a suitable followup by their general practitioner. Secondly, the use of a questionnaire excluded patients with cognitive impairment (>4% of patients in the present study). Note that one cannot exclude that, in this study, slight confusion may have not been identified and may have been ascribed as “general status alteration.” Also, as the collected data is quite subjective, the patient's answers might have been influenced by the attitude of the researcher. Finally, inclusion criteria could overestimate or underestimate the accurate proportion of patients with AF and known as such by their general practitioner. In our study, cases of paroxysmal AF (which nevertheless need anticoagulation) could not be detected in the emergency room and some detected cases of AF might have been de novo or hitherto unknown paroxysmal AF.

Among all of the criteria analyzed in search of characteristics segregating patients who were anticoagulated from patients who were not, only three have been found. The first one is the condition of hospitalization within the 6 months that precede admission in the emergency room. Indeed, the proportion of anticoagulated patients is significantly higher in those who meet this criteria (52% versus 33%). A possible explanation is that hospitalization (whatever the reason) constitutes a prolonged period of medical observation which allows the detection of AF, including paroxysmal forms and the possibility to initiate anticoagulation in an easier way than in ambulatory practice. The second criterion is treatment with digoxin. Patients who were treated with this molecule were more often anticoagulated than subjects who were not (85% versus 55%). A possible explanation is that prescription of digoxin by the general practitioner attests his knowledge of the AF and/or of a closer monitoring of the patient. The third criterion is treatment with >3 different medications, with more such patients being anticoagulated (100% versus 89%). As for the digoxin, this could reflect closer monitoring by the physician.

On the basis of the referenced scores and scales, it was not possible to identify clearly any geriatric characteristic that could be associated with underprescription of AVK in AF. In other words and in accordance with the study by de Breucker et al. [33], no geriatric characteristic seems predictive of the decision by the physician to prescribe or not to prescribe anticoagulation in AF in the elderly. It can however not be excluded that such characteristic might be identified in a larger study. In the present study, 61% of patients with AF were anticoagulated, which is a higher share than the about 40% generally reported in medical literature [16–18]. A potential reason is the very large proportion of subjects included in the study (99%) which are monitored by a general practitioner. On the basis of the CHADS_2_ score alone, 73% of the patients with AF should have been anticoagulated (score ≥ 2). Only 22% of anticoagulated patients had an INR between 2 and 3, which is much lower than the amount of 40 to 58% generally reported in literature [26, 27]. The enrollment of subjects in an emergency room (possibly unstable situation) may have underestimated the proportion of therapeutic INR in a stable situation. Of the 15 anticoagulated patients who were admitted for a trauma or a fall, no severe hemorrhage was diagnosed, even in patients with INR >3 at the time of admission. Some observations may be made about comorbidities and comedications: 33% of patients with AF had heart failure and 60% had hypertension. This share is close to the one reported in the general geriatric population. Hypertension is known to be the most significant factor for AF in the elderly. 18% of patients with AF had previous history of stroke or TIA. In our subject pool, however, this previous history did not appear to influence the decision of the physician to prescribe anticoagulation. Such patients were not more often anticoagulated than others. This is in concordance with literature data on the influence of previous history of stroke on physician's decision to prescribe anticoagulation, with only the hemorrhagic type being known to exert an influence, by discouraging the prescription [1]. On the other hand, polymedication was systematic in our group of anticoagulated subjects: they all took at least 3 different medications, with an average of 7.

We have to recognize two limitations of our study that were due to the methodology used. Firstly, by excluding patients not able to answer the questionnaires of the comprehensive assessment, we couldn't study the impact of cognitive disorders. These are indeed often associated with underprescription of anticoagulation. Secondly, the recruitment in an emergency service required the use of a geriatric assessment that is less exhaustive than the classical “comprehensive geriatric assessment.”

## 6. Conclusion

From this study of consecutive ≥75 years old patients with AF admitted in an emergency room and able to answer a questionnaire, no specific geriatric characteristic could be identified as significantly associated with, thus possibly predictive of, underprescription of AVK treatment. Only very few elements that reflect the influence of the quality of the monitoring on the probability for the patient to be actually anticoagulated could be noted. In accordance with available literature data, the present study confirms the underprescription of AVK in AF in the elderly and the lack of consideration for the CHADS_2_ criteria. This study reports a very weak rate of INR in the therapeutic range, although with some reserves for its interpretation due to the design of study.

As already suggested, underprescription of anticoagulation in patients with AF is presumably due to complex interaction between patient-, physician-, and health care system-related factors [18], where the need, as well as the difficulty, of maintaining the INR level within the therapeutic range probably plays a major role. Could the new generations of oral anticoagulant (anti-Xa and anti-Ila) change this situation is a relevant question for the future.

## Figures and Tables

**Table 1:** Characteristics of the 142 old patients with AF.

	Mean (SD) or %	Median	Min	Max
% anticoagulation	61%			
AVK	50%			
LMWH	11%			
% aspirin alone	16%			
% association therapy				
Sintrom + aspirin	24%			
LMWH + aspirin	5%			
CHADS_2 _(points)	2.4 (1.2)	2.0	1	5
% CHADS_2_ ≥ 2	73%			
Institution (long term care)	31%			
Number of classes of drugs	7.6 (3.2)	7.0	0	20
Ability to manage drug therapy				
Alone	51%			
Alone if prepared	33%			
Not possible	17%			
ISAR (points)	3.4 (1.7)	3	0	6
% ISAR ≥ 1	98%			
% ISAR ≥ 2	84%			
GDS (points)	0.8 (1.4)	0	0	4
% GDS > 1	33%			
% fallers < 6 months	34%			
% weight loss	34%			
% albumin < 3.5 g/100 mL	25%			
Hospitalizations	76%			

AVK: antivitamin K drug; LMWH: low molecular weight heparin; ISAR: identification of senior at risk; GDS: geriatric depression scale.

**Table:** (a) Geriatric characteristics

	Not anticoagulated		Anticoagulated		*P*
	*n*	Mean (SD) or %	*n*	Mean (SD) or %	
Institution (long term care)	56	37.5%	84	26.2%	0.155
Number of classes of drugs	54	6.5 (2.9)	86	8.2 (3.2)	**0.001**
Drug management ability	53	51%	80	50%	0.915
ISAR (points)	56	3.4 (1.7)	86	3.5 (1.6)	0.6173
% ISAR ≥ 1	56	95%	86	98%	0.338
% ISAR ≥ 2	56	82%	86	85%	0.665
GDS (points)	56	0.9 (1.4)	86	0.8 (1.4)	0.619
% GDS > 1	56	38%	86	30%	0.368
% patients who fell < 6 months	50	30%	77	36%	0.459
% weight loss	53	36%	78	32%	0.651
% albumin < 3.5 g/100 mL	47	26%	69	25%	0.913

**Table:** (b) Comorbidities

	Not anticoagulated		Anticoagulated		*P*
	*n*	%	*n*	%	
% hospitalized < 6 months	52	33%	83	52%	**0.030**
Charlson (points)	54	2.4 (1.8)	83	2.6 (1.9)	0.525
Gastric ulcer	54	15%	84	19%	0.522
Liver	54	5%	84	9%	0.671
Hypertension	54	20%	84	25%	0.529
Myocardial infarction	54	13%	84	13%	0.982
COPD	54	13%	84	13%	0.982
Arteriopathy	54	11%	82	15%	0.553
Connectivitis	54	2%	84	2%	0.835
Cancer	54	24%	84	19%	0.053
HIV	54	0%	84	0%	—
Diabetes (not complicated)	56	9%	86	7%	0.671
Diabetes (complicated)	56	4%	86	3%	0.979
Hematological	54	7%	84	4%	0.316

CHADS_2_ score					
CHADS_2_ (points)	56	2.3 (1.1)	86	2.44 (1.3)	0.557
% CHADS_2_ ≥ 2	56	77%	86	70%	0.360
Congestive	54	28%	84	37%	0.267
Hypertension	54	67%	84	55%	0.165
Age	54	100%	84	100%	—
Diabetes	54	13%	84	11%	0.903
Stroke	54	15%	84	23%	0.284

**Table:** (c) ISAR (identification of senior at risk).

	Not anticoagulated		Anticoagulated		*P*
	*n*	%	*n*	%	
>3 drugs	55	89%	86	100%	**0.002**
Need of help before admission	53	68%	80	64%	0.620
Need for help	53	45%	80	40%	0.546
Visual disorders	53	70%	79	75%	0.538
Memory disorders	52	50%	80	42%	0.398
Hospitalized < 6 months	52	33%	83	52%	**0.030**

**Table:** (d) Drugs

	Not anticoagulated		Anticoagulated		*P*
	*n*	%	*n*	%	
Sintrom	54	0%	86	81%	0.000
LMWH	54	0%	86	19%	0.001
Antiaggregants	54	43%	86	20%	**0.004**
Digoxin	54	7%	86	20%	**0.003**
*β* Blockers	56	45%	86	57%	0.150
Flecainide	54	2%	86	1%	0.738
Ca-antagonists	54	20%	86	16%	0.230
IEC	54	35%	86	34%	0.723
Amiodarone	54	11%	86	22%	0.099
ARB	54	11%	86	10%	0.904
NSAI	54	2%	86	2%	0.851

**Table 3:** Univariate analysis of factors associated with the presence of anticoagulation (*n* = 142).

	Odds ratio (IC 95%)	*P*
Institution	0.59 (0.28–1.21)	0.155
% CHADS_2_ ≥ 2 points	0.69 (0.32–1.49)	0.359
% ISAR ≥ 1 point	2.37 (0.45–5.83)	0.338
% ISAR ≥ 2 points	1.12 (0.50–2.96)	0.664
% GDS > 1 point	0.72 (0.35–1.46)	0.368
% fallers < 6 months	1.33 (0.62–2.83)	0.459
% weight loss	0.84 (0.40–1.75)	0.651
% albumin < 3.5 mg/100 mL	0.95 (0.41–2.21)	0.913
% GFR < 40 mL/min	0.92 (0.35–2.41)	0.874

**Table 4:** Mutivariate analysis of factors associated with the presence of anticoagulation Logistic regression *n* = 135, Prob > *χ* ^2^ = 0.0005, and pseudo *R* ^2^ = 0.08.

	OR (IC 95%)	Err St	*P*
Hospitalization < 6 months	2.38 (1.12–5.04)	0.91	**0.023**
Digoxin therapy	5.24 (1.67–16.42)	3.05	**0.004**

Goodness-of-fit test: *P* = 0.0125.
